# The SASOP/PsychMg child and adolescent attention-deficit/hyperactivity disorder guidelines

**DOI:** 10.4102/sajpsychiatry.v31i0.2357

**Published:** 2025-02-25

**Authors:** Brendan Belsham, Linda Kelly, Renata Schoeman

**Affiliations:** 1 Private Practice, Randburg, South Africa; 2 Private Practice, Cape Town, South Africa; 3 Department of Leadership, Stellenbsoch Business School, Bellville; and Private Practice, Bellville, South Africa

## Disclaimer

These guidelines do not purport to provide a comprehensive review of all the literature pertinent to attention-deficit/hyperactivity disorder (ADHD) but aim to provide a set of diagnostic and treatment guidelines relevant to the South African context. They should be utilised in conjunction with other international guidelines. It remains the responsibility of practitioners to maintain a high level of personal knowledge and expertise. For many South Africans, guidelines such as these remain an unreachable ideal because of poor identification and treatment of common mental disorders and limited access to scarce specialist resources. Most South Africans rely on the public healthcare sector and may not have access to treatment options referred to in these guidelines. These guidelines should therefore not be seen as a policy document.

## Process

The South African Society of Psychiatrists’ Special Interest Group (SIG) for ADHD was launched in 2015. The overall objective of the ADHD SIG is to improve the basket of care available to patients with ADHD. This is only possible through a combined and concerted effort of individuals with a special interest in and passion for ADHD to improve knowledge about and funding for the care of individuals with the disorder. One of the specific aims of the ADHD SIG was to develop South African guidelines for the diagnosis and treatment of adult ADHD (published in 2017) and update guidelines for the treatment of ADHD in children and adolescents, initially published by Flischer and Hawkridge in 2013. The SIG tasked Dr Brendan Belsham and Dr Linda Kelly to update the South African guidelines for managing ADHD in children. They requested assistance from Prof. Renata Schoeman because of her experience in academic writing and guideline development. The initial draft of the guidelines was circulated to a core group of local experts for review. These experts represented the SASOP ADHD SIG, the SASOP Child and Adolescent Psychiatry Special Interest Group (CAPSIG) and the Paediatric Neurology and Development Association of Southern Africa (PANDA-SA). They provided written feedback and evidence-based suggestions, which were then incorporated in the final document that was submitted to the SASOP and PsychMg boards for recommendation and ratification.

## Introduction

The concept of hyperactivity in children has been documented in the medical literature since the 18th Century. In 1798, physician and author Sir Alexander Crichton described a mental state much like ADHD, referring to individuals with a ‘disease of attention,’ who refer to themselves as having ‘the fidgets,’ and have ‘the incapacity of attending with a necessary degree of constancy to any one object,’ hallmark symptoms of what we now call ADHD. He was also accurate in other aspects of his portrayal of the condition, such as its early onset and the possibility of improvement with maturity.^1^ The notion that ADHD is a disorder of childhood prevailed until the turn of the century, but rigorous research, including longitudinal studies, has now documented the persistence of symptoms into adulthood in around 65% of individuals.^2^

Attention-deficit/hyperactivity disorder is the most common psychiatric disorder in children, affecting around 5%–7% of the school-going population,^3^ with the prevalence in 3–12-year-olds slightly higher than in 12–18-year-olds (7.6% vs. 5.6%, respectively).^4^ While in childhood there is a clear male preponderance of ADHD, in adult samples, sex differences are more modest or absent.^5^ The prevalence of ADHD does not significantly differ between Africa and the rest of the world.^6^ A local study found the prevalence of ADHD among primary school children of all ethnic populations in the Limpopo province to be 5.5%.^7^ However, in another community-based sample (*N* = 12 447), the prevalence of ADHD was 2.7% – which may be an underestimation as children with predominantly inattentive symptoms were less likely to be referred for screening and assessment.^8^

It is well recognised that ADHD is highly heritable, in fact among the most heritable of all psychiatric conditions. Family studies have identified a 2 to 8-fold increase in the risk for ADHD in parents and siblings of children with ADHD. Various twin and adoption studies have also highlighted the highly genetic nature of ADHD.^9^ Genetic studies suggest that a plethora of genes, each one with a small but significant effect, interact with environmental factors to increase the susceptibility to ADHD.^10^ Some of these environmental factors include prematurity and low birth weight, early deprivation, maternal obesity, childhood exposure to second-hand cigarette smoke and lead contamination.^11^

Attention-deficit/hyperactivity disorder is a costly, chronic disorder with a significant impact on the quality of life of individuals with ADHD and their families. Comorbidity in children with ADHD is prevalent and can range from poor self-esteem and academic difficulties to clinically relevant mental health disorder diagnoses. This contributes to the burden of disease and reduced quality of life of children with ADHD and their families.^12^ Up to 50% of children have at least one additional psychiatric disorder, while 25% have two or more comorbid disorders.^13^

## Diagnosis and clinical characteristics

The accurate diagnosis of ADHD is a crucial factor in the successful management of this common, but often under- or misdiagnosed condition. The diagnosis should ideally be made by a specialist healthcare professional with appropriate expertise and training in diagnosing ADHD, such as a child psychiatrist, a paediatric neurologist or a neurodevelopmental paediatrician. For a diagnosis of ADHD to be made, symptoms according to the Diagnostic and Statistical Manual of Mental Disorders, fifth edition (DSM-5) diagnostic criteria need to be present (Box 1).^14^

BOX 1 Diagnostic and Statistical Manual of Mental Disorders, fifth edition criteria for attention-deficit/hyperactivity disorder.

- A persistent pattern of inattention and hyperactivity-impulsivity that interferes with functioning or development, as characterised by: (1) inattention and (2) hyperactivity and impulsivity:
  1. **Inattention:** Six (or more) of the following symptoms have persisted for at least 6 months to a degree that is inconsistent with developmental levels and negatively impacts social academic and occupational activities:
    Note: The symptoms are not solely a manifestation of oppositional behaviour, defiance, hostility or failure to understand tasks or instructions. For older adolescents and adults (age 17 years and older), at least five symptoms are required:
    - Often fails to give close attention to details or makes careless mistakes in schoolwork, at work or during other activities (e.g. overlooks or misses details, work is inaccurate).
    - Often has difficulty sustaining attention in tasks or play activities (e.g. has difficulty remaining focussed during lectures, conversations or lengthy reading).
    - Often does not seem to listen when spoken to directly (e.g. mind seems elsewhere, even in the absence of any obvious distraction).
    - Often does not follow through on instructions and fails to finish schoolwork, chores or duties in the workplace (e.g. starts tasks but quickly loses focus and easily side tracked).
    - Often has difficulty organising tasks and activities (e.g. difficulty managing sequential tasks; difficulty keeping materials and belongings in order; messy, disorganised work; has poor time management and fails to meet deadlines).
    - Often avoids, dislikes or is reluctant to engage in tasks that require sustained mental effort (e.g. schoolwork or homework; for older adolescents and adults, preparing reports, completing forms and reviewing lengthy papers).
    - Often loses things necessary for tasks or activities (e.g. school materials, pencils, books, tools, wallets, keys, paperwork, eyeglasses and mobile telephones).
    - Is often easily distracted by extraneous stimuli (for older adolescents and adults, may include unrelated thoughts).
    - Is often forgetful in daily activities (e.g. doing chores, running errands; for older adolescents and adults, returning calls, paying bills and keeping appointments).
  2. **Hyperactivity and impulsivity:** Six (or more) of the following symptoms have persisted for at least 6 months to a degree that is inconsistent with developmental levels and negatively impacts directly on social, academic and occupational activities:
    Note: The symptoms are not solely a manifestation of oppositional behaviour, defiance, hostility or a failure to understand tasks or instructions. For older adolescents and adults (age 17 years and older), at least five symptoms are required.
    - Often fidgets with or taps hands or feet or squirms in seat.
    - Often leaves seat in situations when remaining seated is expected (e.g. leaves his or her place in the classroom, in the office or other workplace or in other situations that require remaining in place).
    - Often runs about or climbs in situations where it is inappropriate. (Note: In adolescents or adults it may be limited to feeling restless.)
    - Often unable to play or engage in leisure activities quietly.
    - Is often ‘on the go’, acting as if ‘driven by a motor’ (e.g. is unable to be or uncomfortable being still for an extended time, as in restaurants or meetings; may be experienced by others as being restless or difficult to keep up with).
    - Often talks excessively.
    - Often blurts out an answer before a question has been completed (e.g. completes people’s sentences; cannot wait for his or her turn in conversation).
    - Often has difficulty waiting his or her turn (e.g. while waiting in line).
    - Often interrupts or intrudes on others (e.g. butts into conversations, games or activities; may start using other people’s things without asking or receiving permission; for adolescents and adults, may intrude into or take over what others are doing).
- Several inattentive or hyperactive-impulsive symptoms were present prior to 12 years of age.
- Several inattentive or hyperactive-impulsive symptoms are present in two or more settings (e.g. at home, school or work, with friends or relatives or in other activities).
- There is clear evidence that the symptoms interfere with or reduce the quality of social, academic or occupational functioning.
- The symptoms do not occur exclusively during the course of schizophrenia or other psychotic disorders and are not better explained by other mental disorders (e.g. mood disorder, anxiety disorder, dissociative disorder, personality disorder, substance intoxication or withdrawal).

*Source*: American Psychiatric Association (APA). Diagnostic and statistical manual of mental disorders. 5th ed. c2013^14^

The diagnosis requires the presence of developmentally inappropriate levels of the triad of hyperactive, impulsive and inattentive symptoms for at least 6 months. Symptoms need to occur in two or more different settings (e.g. home and school), should cause impairment in functioning or development across social, academic or occupational settings and at least some symptoms need to have been present before the age of twelve.

Based on these criteria, three types of ADHD are identified:

- Attention-deficit/hyperactivity disorder combined type: If both criteria A1 and A2 have been met for the past 6 months.
- Attention-deficit/hyperactivity disorder predominantly inattentive type: If criterion A1 has been met, but criterion A2 has not been met for the past 6 months.
- Attention-deficit/hyperactivity disorder predominantly hyperactive-impulsive type: If criterion A2 has been met, but criterion A1 has not been met for the past 6 months.

Further distinctions are made about severity:

- Mild: Few, if any, symptoms above those required to make the diagnosis are present, resulting in no more than minor impairments in social or occupational functioning.
- Moderate: Symptoms or functional impairment between ‘mild’ and ‘severe’ are present.
- Severe: Many symptoms above those required to make the diagnosis or several particularly severe symptoms are present or the symptoms result in marked impairment in social or occupational functioning.

### Screening

As ADHD is a common neurodevelopmental disorder that presents in early childhood, ideally screening for ADHD symptoms in children in preschool or early primary school would assist in preventing the consequences of delayed or missed diagnosis; however, the practicality of this in South Africa is limited. It is important to note that the agreed minimally acceptable balance of sensitivity and specificity for screening tools is 0.8. Systematic reviews and meta-analyses of screening tools for ADHD have indicated that most acknowledged tools have good overall diagnostic accuracy, but that a single measure completed by a single reporter is unlikely to have sufficient sensitivity and specificity for clinical use or population screening.^15^

Once children are referred to a specialist, screening for ADHD should occur as part of the psychiatric diagnostic history taking in all patients regardless of the referred complaint. In conjunction with the clinical history, rating scales screening for ADHD are often used. These cannot be used alone to diagnose ADHD; positive screens can prompt full diagnostic evaluations.

In South Africa, the most used screening instruments are the Conners’ Parent Rating Scale (CPRS) and the Conners’ Teacher Rating Scale (CTRS),^16^ the Swanson, Nolan and Pelham Rating Scale (SNAP-IV) for Teachers and Parents,^17^ the Vanderbilt ADHD Diagnostic Rating Scale for teachers (VADTRS) and parents (VADPRS)^18^ and the Copeland Symptom Checklist.^19^

A study comparing a semi-structured interview versus CTRS/CPRS found sensitivity and specificity values to be high for the diagnostic interviews (91.8% and 70.7%, respectively), while sensitivity of the CTRS/CPRS was relatively high (83.5%), but specificity was poor (35.7%).^8,20^

A systematic review of tools for the diagnosis of ADHD in children and adolescents, indicated the sensitivity of the SNAP-IV for parents as ranging from 96% to 100%, with a 4%–38% specificity in clinical samples. The SNAP-IV for teachers sensitivity ranged from 40% to 97%, with specificity ranging from 26% to 71%.^21^ The SNAP is therefore sensitive for picking up ADHD symptoms, but not accurate in distinguishing whether the symptoms are specific to ADHD or not. In general, parent scores showed a stronger association with ADHD than teacher scores. Teachers’ scores were specifically poor in detecting inattentiveness.^22^

The VADTRS and VADPRS are freely available in the public domain, making it an accessible screening instrument. Studies indicated Cronbach’s alpha reliability for the VADPRS of 0.93–0.95 in clinical study samples, while the reliability for the VATRS ranged from 0.9 to 0.92 in school screens and 0.93–0.94 in clinical study samples.^19^ A small South African study of 100 children between the ages of 6 and 12 found acceptable reliability of the VADRS with all subscale alphas above 0.7. However, the study found that contextual factors impact significantly on how the child was rated and need to be considered when interpreting findings.^23^

Rating scales are also useful for monitoring intervention responses and comparing them to baseline symptoms.

### Clinical evaluation

The clinical interview remains the cornerstone of the assessment process in diagnosing ADHD. In assessing a child, a full clinical and psychosocial history should be obtained from parents or caregivers, and the child or adolescent themselves where appropriate. Collateral information from schools, such as report cards and rating scales completed by teachers, is also invaluable. Collateral information and assessment reports from therapists involved with the patient, such as educational psychologists, occupational therapists (OT), optometrists and speech and language pathologists, should also be reviewed. Certain conditions or circumstances may point to an increased risk for ADHD, such as premature birth or a complicated neonatal course, neurodevelopmental disorders (including intellectual disability, autism spectrum disorders (ASD), specific learning disabilities, tic disorders and congenital syndromes), the presence of oppositional defiant symptoms or behavioural challenges, mood and anxiety disorders, epilepsy, traumatic brain injury, substance abuse, the presence of ADHD in a close relative and children in foster or alternative care arrangements.^24,25^

### Medical, laboratory and other special investigations

A thorough medical history and physical examination should be completed to exclude any causative or comorbid medical disorders confounding the diagnosis. It is also important to document baseline blood pressure, pulse rate, height and weight, which should be monitored at follow-up consultations.^24^

Most patients with ADHD do not require laboratory investigations, which should be reserved to eliminate suspected pathology (e.g. iron deficiency) or for safety monitoring with the use of certain medications (e.g. liver function tests with the use of atomoxetine [ATX]). An electrocardiogram (ECG) may be required if there is a history or family history of significant cardiac pathology, particularly congenital cardiac defects or cardiac surgery. Visual and auditory screening should be done with a referral to optometrists or audiologists for further investigation when indicated. Neurological symptoms may require referral to a neurologist. Routine electroencephalogram (EEG) is not recommended as part of the diagnostic workup for ADHD. If epilepsy is suspected, an EEG should be performed. Although extended-length EEGs have a higher likelihood of detecting interictal phenomena, cost and convenience factors should be considered.^26^ Despite the United States Food and Drug Administration’s approval of the NEBA (Neuropsychiatric EEG-based Assessment Aid) system to evaluate theta and beta wave ratios, studies have not found this to be a reliable method to diagnose ADHD and the use of EEG biomarkers for diagnostic purposes is not supported as yet.^27,28^

Although neuroimaging, brain mapping and event-related potentials have detected structural and functional differences in ADHD research studies, there are currently no indications for these costly and inaccessible investigations in clinical practice.^11,25^

### Psychometric evaluation

There are currently no neuropsychological tests for ADHD with sufficient sensitivity and specificity to serve as an individual diagnostic test.^11^ Neuropsychological and psychoeducational evaluations should be interpreted within the broader clinical evaluation and are not in themselves diagnostic. These tests can be useful where comorbidity and diagnostic uncertainty exist and to individualise interventions. Computerised assessments such as the Test of Variable Attention (TOVA),^29^ MOXO d-CPT^8,30^ and QbTest^31^ have not yet been established as useful in children and adolescents.

### Comorbidity

Comorbid conditions are very common with ADHD and often complicate the clinical presentation. Fifty to ninety per cent of children have at least one comorbid condition, while 50% have two or more comorbid conditions.^25,32^ Follow-up studies of children with ADHD and comorbidity show poorer outcomes and greater social, emotional and psychological difficulties.

Comorbid conditions are either current, episodic conditions – the most frequent in childhood being disruptive behavioural disorders (e.g. oppositional defiant disorder, conduct disorder), anxiety disorders (e.g. generalised anxiety disorder, social anxiety and obsessive-compulsive disorder) and mood disorders (e.g. depression and bipolar disorder). Others represent lifespan disorders, such as specific developmental disorders of language, learning and motor development, ASD and intellectual disability.^25,32^

Attention-deficit/hyperactivity disorder does not have any one symptom that is pathognomonic for the condition and overlap with other disorders can be significant. Comorbid conditions differ according to developmental stage and change over time. In early childhood, the most common comorbid disorders are oppositional defiant disorder, language disorders and anxiety disorders. In middle school children, anxiety and tic disorders become more common. In adolescence, mood disorders and substance use disorders become more prevalent.^25^

Oppositional defiant disorder and conduct disorder are common comorbidities with ADHD in childhood and adolescence, with 30%–50% fulfilling the criteria for one of these two conditions. Other externalising disorders such as disruptive mood dysregulation disorder and intermittent explosive disorder also occur relatively commonly. These comorbidities are associated with greater functional impairment, higher rates of learning disabilities and academic problems, behavioural problems, aggression and later, substance use and delinquent behaviour.^33^ Treatment should be multimodal, including medication, parent management training, cognitive behavioural therapy and family interventions.

Anxiety disorders and mood disorders also commonly co-occur with ADHD. Anxiety disorders are seen in 15%–35% of children with ADHD and depressive disorders in 20%–50%.^34^ For bipolar disorder, the rates vary widely across studies.^35^ The most impairing condition should be treated first, though both conditions usually require treatment. The comorbid condition may be aggravated by the others’ treatment, thus slow titrations and careful monitoring are necessary. The demoralisation and low mood resulting from living with ADHD are differentiated from depressive disorders that have an independent and distinct course from ADHD. In bipolar disorder, one should stabilise the mood first and then treat the ADHD.^36^ When these two disorders co-occur, patients have poorer global functioning and greater symptom severity than for either alone.^37^

Tic disorders including Tourette syndrome are highly comorbid with ADHD, with prevalence rates of 55%–90%. The presence of tics is not a contraindication to using stimulants for ADHD, but careful monitoring is required. If stimulants do increase tics in a particular individual, then ATX should be considered, as it has a lower potential to aggravate tics.^38^ The co-occurrence of obsessive-compulsive disorder and ADHD further increases the risk for tic disorders and Tourette syndrome.^39^

In previous editions of the DSM, the diagnosis of ADHD and ASD was mutually exclusive. However, since the publication of DSM-5, there has been a substantial increase in the prevalence of these two disorders, which can now be diagnosed in combination. Thirty to seventy per cent of patients with ASD meet the criteria for ADHD, while many individuals with ADHD display social deficits and ASD-like symptoms.^40,41,42^ Children with combined ADHD and ASD have more severe symptoms across most domains including sleep disturbances.^43^ Trials have shown that treating ADHD with comorbid ASD is effective, but side effects may be more prevalent, and medication should be started at lower doses and titrated carefully.^44^ Attention-deficit/hyperactivity disorder is two to three times more common in children with developmental disabilities, borderline IQ and intellectual disabilities. Around 45% of children with ADHD have a specific learning disorder, including disorders of reading, written expression, auditory processing, visual processing and processing speed. Children with either ADHD or a learning difficulty should be screened for the other condition, – as outcomes are significantly improved if both conditions are receiving necessary intervention.^45^

Sleep disturbance occurs in around 50% of individuals with ADHD, most commonly initial insomnia. There is growing evidence that individuals with ADHD may be more susceptible to circadian rhythm disruptions.^46^ Sleep problems may also aggravate ADHD symptoms, and pharmacological interventions for ADHD may impact sleep. Although methylphenidate does not alter sleep architecture, it may delay sleep onset, reduce sleep efficiency and shorten total sleep duration.^47^ Behavioural sleep interventions and melatonin are considered effective interventions for sleep disturbances in the child and adolescent populations.^48,49^

Developmental and acquired brain injuries are also associated with ADHD-like symptoms. The association between foetal alcohol syndrome and ADHD is strong and can be particularly difficult to manage.^50^ Children with moderate to severe brain injury have up to a 48% chance of secondary ADHD and should receive treatment with close monitoring for side effects.^51^

Comorbidity between epilepsy and ADHD is common. Identification of ADHD in patients with epilepsy is difficult because the symptoms and management related to epilepsy can directly contribute to attention deficits.^52^ Twenty to forty per cent of children with epilepsy have ADHD, with significant ADHD symptoms present in up to 82% of children before the onset of the first seizure. Children with ADHD have a two to fourfold higher risk for epilepsy when compared to controls (1.2%–14% of children).^53^ Vigilance is important, especially in female patients and those with the predominantly inattentive subtype. Both conditions require rigorous management to improve clinical outcomes and quality of life. Anticonvulsants should be chosen with a view to minimising behavioural and cognitive side effects. There is no strong evidence to suggest that psychostimulants increase the severity or frequency of seizures in patients with stable epilepsy.^54,55^

The prevalence of ADHD in eating disorders (such as anorexia nervosa and bulimia nervosa) is around 11%, while roughly 20% of children with ADHD also develop eating disorders.^56^ Screening for ADHD should be part of an eating disorder history and vice versa.^57^ There is also a bidirectional relationship between ADHD and obesity.^58^ Attention-deficit/hyperactivity disorder and ASD are more prevalent in children and adolescents with obesity than in those without obesity.^59^ Suggested mechanisms include impulsive eating, common genetic underpinnings involving the dopaminergic system and obesity-related risk factors such as sleep-disordered breathing.^60^

Attention-deficit/hyperactivity disorder increases the risk for an elimination disorder (enuresis and encopresis) two to three times. Elimination disorders should be investigated and managed, but recent studies have found that treatment of ADHD with both psychostimulants and ATX can result in an improvement in enuresis and encopresis.^61^

Time spent using digital media for leisure purposes has increased exponentially in the last decade among children and adolescents, and social media use has been found to be just as addictive as gaming. Longitudinal studies have looked at the direction of association between digital media use and ADHD and have revealed that excessive digital media use (EDMU) is a risk factor for the onset of ADHD symptoms, while ADHD predisposes to EDMU. Furthermore, ADHD and EDMU mutually aggravate each other over time.^62^ A recent study showed that 44% of children and adolescents with ADHD met the criteria for internet gaming disorder (IGD), compared to 9.5% of controls. Attention-deficit/hyperactivity disorder patients with IGD presented with greater severity and impairment, more severe ADHD symptomatology, more internalising symptoms (particularly withdrawal/depression and socialisation problems), and a greater likelihood of other addictive behaviours.^63^

Therefore, from a clinical perspective, it is important for practitioners to educate parents and children about how EDMU may aggravate ADHD symptoms. Various bodies, such as the American Academy of Pediatrics have published guidelines for recreational screen time in children (www.healthychildren.org).

## Treatment

Good long-term clinical care is contingent upon adequate patient education about the nature of the disorder and the effects of treatment. Once the diagnosis has been confirmed, clinicians should provide sufficient information to empower families to make informed decisions about treatment options. While published guidelines such as these are important, it must be stressed that a strictly algorithmic approach to treating ADHD is not advised, and management should be holistic, multidisciplinary and tailored to meet the individual needs of patients and their families.

Although pharmacotherapy plays a primary role in the treatment of ADHD, psychosocial interventions form an integral part of a comprehensive, multi-modal management approach for children with ADHD. This multidisciplinary approach is also encapsulated in various international guidelines that recommend psychosocial treatments as complementary to psychopharmacological interventions to provide support, improve acceptance of diagnosis and treat comorbidities and residual symptoms.^24,25,64^

### Factors to consider in choosing attention-deficit/hyperactivity disorder medication

A detailed history outlining previous trials of medication is important to facilitate rational decision-making around medication choice. However, it must be stressed that an individual child may respond very differently to a given medication at different stages of development. Very young children may tolerate the shorter-acting preparations better than the longer-acting ones because of the reduced risk of appetite suppression and insomnia. Of particular importance is the management of preschool children with ADHD. It is well recognised that children below the age of five are more vulnerable to the side effects of ADHD medications. Therefore, it is appropriate to implement environmental modifications and parent management training before considering medications in this age group.^24^

Comorbid conditions must be managed concomitantly with ADHD and decisions regarding which to treat first depend on the severity of each condition. The more severe condition should generally be addressed first while balancing functional impairment and possible worsening of the comorbid condition.^65,66^ It is highly recommended that ADHD with comorbidity be managed at the specialist level.

Access to medication is not a given. Most South Africans are reliant on the state healthcare sector and thus do not have access to the full range of locally available medications. Even in the private sector, stock shortages periodically impact medication choice. Many families struggle to afford the ongoing costs of ADHD medication, and increasingly, more than one individual in a family may be on treatment. Most medical aids do not offer chronic benefits for ADHD and its medications, and most South African families are not covered by medical insurance.^67^

There is increasing interest in the use of pharmacogenetic testing to guide medication choice for ADHD. However, to date, effect sizes have been small, and few consistent findings have been reported. Given the poor adherence to ADHD medications, perhaps a more appropriate target for pharmacogenetic studies is tolerability. Because many common adverse events are dose-related, pharmacogenetic studies associated with inter-individual variability in drug metabolism may be helpful, such as CYP2D6 for amphetamines (AMP) and ATX and carboxylesterase-1 gene (CES1), which is the sole enzyme involved in methylphenidate (MPH) metabolism.^68^ While this emerging field of precision medicine holds promise, there remains no substitute for a thorough clinical assessment and decision-making process that incorporates the factors outlined in this section.

### Pharmacological treatment

Medication remains the cornerstone of treatment for most children and adolescents with ADHD.^24,45,64^ As with all pharmacological treatments in medicine, the risk–benefit ratio should be considered before initiating any medication. The high morbidity of ADHD makes it essential to consider the risks of not treating the condition, which has been associated with decreased social, educational, and vocational functioning, and poorer self-care, as well as higher rates of accidental injury.^69^ From a pharmacoeconomic perspective, it is also now well established that pharmacotherapy of ADHD is cost-effective compared to no treatment or behavioural therapy.^70^

Medications used in the treatment of ADHD in children include stimulants (MPH and AMP) and non-stimulants (ATX, alpha2-adrenoceptor agonists [clonidine and guanfacine] and tricyclic antidepressants [TCAs]). Enhancement of dopaminergic and noradrenergic neurotransmission in the prefrontal cortex is considered critical to the therapeutic efficacy of ADHD medications.^71^

#### Stimulants

Stimulants are the best-studied pharmacological treatment for ADHD across the lifespan. Methylphenidate and AMP have similar effects, with an average response rate of 70%.^72^ In addition to reducing the core symptoms of ADHD, stimulants have been shown to improve associated features such as defiance, academic performance, social functioning, as well as fine and gross motor functioning.^11^ These effects appear to be dose dependent and are evident across multiple settings.^24,25,64^ Internationally, most treatment guidelines recommend one of the stimulants as the first-line pharmacological treatment for ADHD.^24,25,64^ However, it must be stressed that treatment choice depends on a variety of factors, which may vary between individual patients and the choice of medication ultimately resides with the prescribing doctor in collaboration with the patient and his or her family. Of the two stimulant groups, MPH would generally be used before AMP in South Africa because of greater prescriber experience and availability of the former in both public and state sectors. See Table 1 for stimulants available in South Africa.

**TABLE 1:** Stimulant medications available in South Africa.

Substance	Trade name	Duration of action	Formulation	Starting dose	Dosing interval	Maximum daily dose
**Methylphenidate immediate release**	Ritalin^®^ Douglas MPH^®^ Biotech MPH^®^	4 h	10 mg tablet	5 mg daily at breakfast	Two or three times daily, 4 h apart	60 mg
**Methylphenidate extended release**	Ritalin LA^®^	6 h–8 h	Spheroidal oral drug absorption system (SODAS) 60% immediate release: 40% extended release 10 mg, 20 mg, 30 mg and 40 mg capsules	10 mg daily at breakfast	Once daily	60 mg
	Medikinet MR^®^	6 h–8 h	50% immediate release: 50% extended release 5 mg, 10 mg, 20 mg, 30 mg and 40 mg capsules			
	Concerta^®^ Neucon^®^ Unicorn MPH^®^, Mefedinel^®^	10 h–12 h	Osmotic release system (OROS): 22% immediate release: 78% extended release 18 mg, 27 mg, 36 mg and 54 mg hard-shelled capsules, must be swallowed whole	18 mg daily at breakfast	Once daily	Children: 54 mg Adolescents: (13–18 years) 72 mg
	Contramyl XR^®^	10 h–12 h	Multi-unit particulate system 18 mg, 27 mg, 36 mg and 54 mg tablets			
	Radd^®^	10 h–12 h	Hydrophilic Matrix release system			
	Acerta^®^	10 h–12 h	Extended-release film-coated tablets			
**Dexamfetamine sulphate**	Amfexa^®^	6 h–7 h	5 mg and 10 mg breakable tablets	5 mg daily at breakfast	Once or twice daily, 6 h apart	20 mg (up to 40 mg in rare cases)
**Lisdexamfetamine†**	Vyvanse^®^	14 h	30 mg, 50 mg and 70 mg capsules	30 mg daily with breakfast	Once daily with breakfast	70 mg

MPH, methylphenidate.

† , not registered for use in children below 13 years.

It is important to note that both classes of stimulant medication have similar efficacy and tolerability profiles at the population level. However, at the individual level, patients may respond to or tolerate one class better than the other.^73,74^ It may therefore be appropriate for an adequate trial of *both* classes of stimulants to be given before engaging in a trial of non-stimulant alternatives. Attention-deficit/hyperactivity disorder is a highly heterogeneous condition which affects individual patients differently. Some children may be primarily impaired in the classroom, whereas others may be severely impaired at home and less so at school. The duration of action of the chosen medication is thus an important consideration. Long-acting preparations are usually preferred as they diminish the need for multiple intraday dosing and therefore augment adherence, symptom coverage and treatment response.^75^

However, longer-acting stimulants increase the risk of certain side effects such as appetite suppression and insomnia, especially in younger, treatment-naive children, in whom initiation with a shorter-acting agent may be preferable. Combinations of immediate and extended-release preparations may sometimes be appropriate, for example, a child at boarding school who is required to do homework after 6 pm may require a ‘top-up’ of a short-acting stimulant in the late afternoon or early evening. Compared to immediate-release psychostimulants, the use of long-acting stimulants may diminish diversion and rebound and are often associated with better tolerability.^76^

There is very little correlation between body weight and dosage of the stimulant medications. The dosage should be gradually titrated against clinical response and side effects until an optimum response is attained.^25^ In practice, this may result in dosages that are above the maximum published dosages according to the package insert. In such cases, patients should be under specialist care and frequent monitoring of side effects should be undertaken.

#### Non-stimulants

Atomoxetine has a slightly lower effect size (overall response rate) compared to psychostimulants.^77^ The recommended dose of ATX is 1.2 to 1.8 mg/kg/day.^78^ Atomoxetine may be considered for patients who experience significant side effects or who have had suboptimal responses to stimulants.^79^ It may also be used in combination with stimulant medication in patients whose response to the latter has been suboptimal as there may be synergistic effects in some patients when these two agents are used together. However, the concurrent use of a stimulant and ATX should be under the care of a specialist and blood pressure and pulse should be regularly measured because of the catecholaminergic effect of both medications. Atomoxetine may also be prescribed when stimulant agents are contraindicated or where there is a high risk of stimulant misuse.^80^ In patients with significant comorbid anxiety, ATX may also be preferable as it has potential anxiolytic benefits, most notably in social anxiety, and may be less likely than stimulant medications to aggravate anxiety.^81^

Guanfacine is another non-stimulant for ADHD but not available in South Africa. Other agents used off-label (with lower efficacy profiles and more severe side effects) include clonidine (Menograine^®^) and imipramine (Tofranil^®^). There is no evidence for the use of bupropion or modafinil in the treatment of children and adolescents with ADHD. Atypical antipsychotics are among the agents used for comorbidities commonly seen with ADHD, often in combination with other agents. These medications are generally reserved for treatment-resistant cases and require specialised care.

### Monitoring of treatment

It is important to clearly identify all ADHD-related impairment and the treatment goals at the onset of treatment. Regular re-evaluations of the efficacy and tolerability of treatment for accurate monitoring of progress are needed. It is also important to systematically identify other potential causes of impaired functioning in a patient. Sleep deprivation, poor nutrition, excessive screen time, lack of routines, psychosocial stressors and comorbid disorders can affect outcomes and should always be considered when assessing the patient’s condition and when measuring clinical response to treatment.^25^

After initiation of treatment, the patient should be seen within 4–6 weeks to assess the response to treatment and possible side effects. Thereafter, follow-up intervals are at the doctor’s discretion, but legally cannot be longer than six months apart, because of the high schedule classification of the medications involved. Monitoring requires feedback from the child, his or her parents, the schoolteacher and any therapists who may be involved with the care of the child. To this end, rating scales may be useful.^25^ Height and weight should be monitored because of the appetite-suppressing effects of ADHD medications and potential growth concerns. In children at risk for cardiovascular side effects, blood pressure and pulse should also be monitored.

Interruption in treatment is not recommended. Atomoxetine must be given continuously, inclusive of weekends and holidays, and the same is generally advisable for stimulant medications. Medication may be reduced, temporarily interrupted or completely stopped if the patient experiences unacceptable side effects, ADHD symptoms have improved sufficiently or because of a lack of response. While most children with ADHD will continue to experience symptoms into adolescence and adulthood, there are some whose symptoms remit or in whom the acquisition of coping mechanisms renders the symptoms less impairing.^82^ In such children, a trial without medication may be warranted, in collaboration with the child, their parents and teachers. This process may be particularly important in adolescence when the child’s emerging autonomy should be accommodated. A trial without medication, or using a reduced dosage, should generally not be done at times of transition, such as the beginning of a school year with a new teacher, or at vulnerable times, such as the lead-up to examinations. If there are concerns about a child’s growth or weight, then drug holidays may be appropriate to allow for catch-up growth and/or weight gain.^83^ In some cases, medications are stopped for non-clinical reasons such as stigma, lack of financial coverage or lack of access to healthcare.^64^

### Non-pharmacological interventions for attention-deficit/hyperactivity disorder

Attention-deficit/hyperactivity disorder has a heterogeneous group of causal and contributing factors, including biological, psychological and environmental elements.^84,85^ This increased recognition of factors contributing to ADHD expands possible techniques and outcomes for intervention beyond purely pharmacological methods, which do not target non-biological contributors that exacerbate the condition. The availability of non-pharmacological treatments is increasing, but the literature on these treatments using gold-standard designs is not keeping pace. Difficulties in creating blinded and unbiased studies, as well as comparison to control groups and accounting for placebo effects, are some of the methodological issues encountered when evaluating non-pharmacological treatments for ADHD.^86^

#### Psychoeducation and therapy

Psychoeducation focussed on ADHD is a vital component of the ongoing management of the condition. This should include the guardians/caregivers and the child or adolescent if developmentally appropriate. The focus of psychoeducation should be on information about symptoms of ADHD, treatment options, possible side effects of medication, compliance with medication and discussions around strategic treatment interruption. Other lifestyle factors such as supplements, diet, physical activity, screen time, sleep and discipline strategies may also form part of ongoing psychoeducation. Psychoeducation may also extend to other individuals involved in the child’s life such as teachers, tutors and caregivers.^24,25^

Behavioural therapy principles including social learning theory and other cognitive techniques form the basis of parent counselling and skills training and may take place over multiple sessions. Other formats include group-based parent training, teacher training interventions and child training interventions. Topics to be included are organisational skills, social skills, problem-solving and cognitive restructuring.^87^

Systematic reviews of randomised control trials found small effects on academic outcomes, small to medium effects on parenting and medium to large effects on organisational skills.^87,88^ Behaviour therapy has a consistently strong effect on the broader well-being and outcomes in children and adolescents as part of the management of ADHD.^88^ However, research investigating the efficacy of these interventions is complex, and the literature is often difficult to interpret because of the heterogeneity of interventions and lack of adequate blinding in many studies. Therefore, the effect on core ADHD symptoms remains inconclusive.^87,89,90,91^

Randomised controlled trials of mindfulness-based interventions compared with no treatment have shown some improvement in ADHD symptoms at 6-month follow-up.^92,93^ However, other studies have demonstrated positive effects on child emotional regulation, but not on ADHD symptoms. The literature is growing but currently, more evidence is needed before these interventions could be recommended ahead of medication and behavioural therapy.^87,94^

#### Cognitive training, neurofeedback and brain stimulation

The large variation in targeted areas and frequency of dosing make cognitive training difficult to appraise as an evidence-based treatment. If only blinded studies are considered, no effect of cognitive training and neurofeedback on ADHD symptoms is seen. High costs and low efficacy of cognitive training, even in game-like form, do not support this as an intervention.^95,96,97^

There has been an increasing interest in and the use of computer-based cognitive training (CBCT) as a treatment of ADHD, with some empirical support in recent years from controlled studies.^98^ However, a review of the current evidence found that the effects were stronger for unblinded measures, while controlled trials with blinded measures could not support any significant effect.^99^

Trigeminal nerve stimulation (TNS) and transcranial magnetic stimulation (TMS) have been advocated for children with ADHD but cannot yet be recommended because of insufficient evidence.^87,100,101^

#### Allied therapies

Children who have difficulties in coordination, both fine and gross motor function as well as visual motor integration problems may benefit from referral to an OT. Sensory processing disorder is also a common comorbid finding, which might necessitate occupational therapy. Speech and language delays and articulation disorders require referral to speech and language therapists for assessment and treatment. Children with motor coordination disorders may require referral to a neurodevelopmental physiotherapist.^12^

#### Exercise

Exercise as an intervention for ADHD is appealing, with positive effects on cognitive, motor and socio-emotional development, as well as low cost and safety aspects. Exercise has been shown to enhance dopamine and monoamine neurotransmission, brain-derived neurotrophic factor (BDNF) levels, brain oxygenation and cell metabolism and has been postulated to support brain structural growth and functional neurocognitive development.^102^

In a recent meta-analysis, exercise was found to improve attention, motor skills and executive function in ADHD children compared to controls but did not significantly improve hyperactivity, depression, aggression and social problems.^102^ This contrasts with a previous meta-analysis which found that exercise improved anxiety, depression, aggressive behaviour, mindset and social problems in children with ADHD.^103^

Because of a large difference in study designs and outcome measures, there is still insufficient data to recommend a specific type of exercise, intensity, duration and frequency. Immediate cognitive effects of exercise may not equate to longer-lasting effects of repeated exercise on ADHD symptoms.^87^ Therefore, physical activity as an ADHD intervention remains inconclusive, but the broader benefits of exercise as part of physical and cognitive health seem clear.

#### Dietary interventions

Dietary interventions for ADHD are safe but can be laborious to implement and adequate nutrition needs to be ensured. Such interventions have included eliminating artificial food colouring, additives, salicylates and antigenic foods (oligo-antigenic diet), and the Dietary Approaches to Stop Hypertension (DASH) diet, the latter two demonstrating some evidence in improving ADHD symptoms.^104,105,106,107,108^ There are currently no blinded randomised controlled trials and the general recommendation is to follow a healthy, Mediterranean-type diet. More rigorous studies need to be conducted before firm recommendations can be made.^106,107^

### Complementary and alternative medications

Complementary and alternative medicine (CAM) refers to health and wellness products and techniques not presently considered to be part of conventional Western medicine.^109^ Complementary medicine is used along with conventional medicine, while alternative medicine refers to approaches instead of conventional medicine. Complementary and alternative medicine includes mind-body medicine (such as meditation, acupuncture and yoga), manipulative and body-based practices (such as massage therapy and spinal manipulation) and natural products (such as herbs and dietary supplements). Integrative medicine purports to combine the best of conventional medical care with the best of evidence-based CAM.

The World Federation of ADHD International Consensus Statement reiterates that non-medication treatments for ADHD are less effective than medication treatments but are frequently useful to help symptoms that remain after the medication has been optimised.^11^ Omega-3 fatty acid supplementation demonstrated small to moderate improvements in ADHD symptoms in three meta-analyses.^110,111,112^ However, there is no evidence of any effect of omega-3 fatty acid supplements on parent- or teacher-rated emotional lability symptoms or oppositional symptoms.^113^ Barragan et al. have also demonstrated that augmentation of stimulants with omega supplementation allowed for a dose reduction of stimulants.^114^

Recent studies highlighted the role of micronutrients and vitamins in ADHD management although the findings remain controversial. Vitamin D supplementation (if deficient) has some benefit, but no evidence was found for supplementation with nutrients such as zinc, vitB12, iron and folate.^115^

Although many individuals, with and without ADHD, report benefits from ‘brainsmart foods’ such as bacopa monnieri, mentat, zinc, L-carnitine, vitamin B6, magnesium, ginkgo biloba, ginseng, Celastrus paniculata, passionflower, acetyl L-carnitine (ALCAR), dimethylethanolamine (DMAE), L-theanine, DHA (dosahexaenoic acid), citicoline, curcumin, phosphatidylserine, vincopetine, L-alpha-glycerylphosphorylcholine and huperzine A, formal studies of the effectiveness of these agents are lacking. At present, there is no consistent evidence from randomised controlled trials for the use of any food supplements, and the use of these products, especially in children, should not be recommended.^116^

### Transitioning from child and adolescent to adult services

It is now well established that ADHD continues into adulthood in most affected individuals and continues to cause significant impairment.^80^ Thus the transition of care between child and adult services must be carefully managed to avoid patients being lost to treatment at this vulnerable juncture. This process will necessarily differ between the public and private sectors but should involve several key components, including education for patients and their families about the persistence of the condition into adulthood, gradual preparation for the child and their parents, effective communication between child and adult services/ practitioners, clear information on available services and how to access them and, where possible, a period of parallel care.^117^

## Summary

Attention-deficit/hyperactivity disorder is a common neurodevelopmental disorder of childhood, persisting across the lifespan. It is associated with significant morbidity and economic burden. A comprehensive diagnostic assessment and diagnostic certainty prior to initiating drug treatment are crucial. Effective treatments are available, leading to a significant reduction in impairment and improvement in quality of life for both patients and their families. It is hoped that these guidelines will raise awareness of the plight of children and adolescents with ADHD, assist practitioners in diagnosing and managing ADHD, and inform funders about the importance of comprehensive, multidisciplinary care in improving outcomes across the lifespan.
